# Metabolic profiling of triple-negative breast cancer cells reveals metabolic vulnerabilities

**DOI:** 10.1186/s40170-017-0168-x

**Published:** 2017-08-22

**Authors:** Nathan J. Lanning, Joshua P. Castle, Simar J. Singh, Andre N. Leon, Elizabeth A. Tovar, Amandeep Sanghera, Jeffrey P. MacKeigan, Fabian V. Filipp, Carrie R. Graveel

**Affiliations:** 1 California State University, Los Angeles, 5151 State University Drive, Los Angeles, CA 90032 USA; 2 Van Andel Research Institute, 333 Bostwick Ave, NE, Grand Rapids, MI 49503 USA; 3 Systems Biology and Cancer Metabolism, Program for Quantitative Systems Biology, University of California Merced, 2500 North Lake Road, Merced, CA 95343 USA; 4 College of Human Medicine, Michigan State University, 15 Michigan St. NE, Grand Rapids, MI 49503 USA

**Keywords:** Triple-negative breast cancer, Metabolism, Rate-limiting enzymes, Receptor tyrosine kinase, Tyrosine kinase inhibitor, Metabolic inhibitor

## Abstract

**Background:**

Among breast cancers, the triple-negative breast cancer (TNBC) subtype has the worst prognosis with no approved targeted therapies and only standard chemotherapy as the backbone of systemic therapy. Unique metabolic changes in cancer progression provide innovative therapeutic opportunities. The receptor tyrosine kinases (RTKs) epidermal growth factor receptor (EGFR), and MET receptor are highly expressed in TNBC, making both promising therapeutic targets. RTK signaling profoundly alters cellular metabolism by increasing glucose consumption and subsequently diverting glucose carbon sources into metabolic pathways necessary to support the tumorigenesis. Therefore, detailed metabolic profiles of TNBC subtypes and their response to tyrosine kinase inhibitors may identify therapeutic sensitivities.

**Methods:**

We quantified the metabolic profiles of TNBC cell lines representing multiple TNBC subtypes using gas chromatography mass spectrometry. In addition, we subjected MDA-MB-231, MDA-MB-468, Hs578T, and HCC70 cell lines to metabolic flux analysis of basal and maximal glycolytic and mitochondrial oxidative rates. Metabolic pool size and flux measurements were performed in the presence and absence of the MET inhibitor, INC280/capmatinib, and the EGFR inhibitor, erlotinib. Further, the sensitivities of these cells to modulators of core metabolic pathways were determined. In addition, we annotated a rate-limiting metabolic enzymes library and performed a siRNA screen in combination with MET or EGFR inhibitors to validate synergistic effects.

**Results:**

TNBC cell line models displayed significant metabolic heterogeneity with respect to basal and maximal metabolic rates and responses to RTK and metabolic pathway inhibitors. Comprehensive systems biology analysis of metabolic perturbations, combined siRNA and tyrosine kinase inhibitor screens identified a core set of TCA cycle and fatty acid pathways whose perturbation sensitizes TNBC cells to small molecule targeting of receptor tyrosine kinases.

**Conclusions:**

Similar to the genomic heterogeneity observed in TNBC, our results reveal metabolic heterogeneity among TNBC subtypes and demonstrate that understanding metabolic profiles and drug responses may prove valuable in targeting TNBC subtypes and identifying therapeutic susceptibilities in TNBC patients. Perturbation of metabolic pathways sensitizes TNBC to inhibition of receptor tyrosine kinases. Such metabolic vulnerabilities offer promise for effective therapeutic targeting for TNBC patients.

**Electronic supplementary material:**

The online version of this article (doi:10.1186/s40170-017-0168-x) contains supplementary material, which is available to authorized users.

## Background

### Triple-negative breast cancer

Triple-negative breast cancer (TNBC) accounts for 15–20% of invasive breast cancers. TNBC is characterized by the lack of estrogen receptor (ER) and progesterone receptor (PR) expression and human epidermal growth factor receptor 2 (HER2) amplification. TNBCs are associated with advanced stage at diagnosis and poorer outcome compared to other breast cancer subtypes [1]. Characteristic TNBC clinical features include a peak in recurrence risk within the first 3 years, a weak relationship between the tumor size and lymph node metastasis, and a peak of cancer-related death in the first 5 years [2]. At the molecular level, TNBC has significant overlap with the basal-like subtype as approximately 80% of TNBCs are classified as basal-like [1]. Currently, TNBCs are treated with cytotoxic combination chemotherapy. Even though TNBC patients have significantly higher rates of pathologic complete response compared to non-TNBC, TNBC patients have decreased 3-year progression-free survival and overall survival rates [1, 3]. Hence, there is a vital need for a comprehensive understanding of the molecular basis of TNBC progression and emerging treatment approaches.

TNBC is a highly heterogeneous disease at the molecular level, and this heterogeneity likely underlies the variable treatment responses in patients. Recent studies involving comprehensive gene expression analysis revealed extensive molecular heterogeneity within TNBC cases and identified four to six distinct molecular TNBC subtypes [4, 5]. These subtypes have unique expression signatures and ontologies and are defined as basal-like, mesenchymal and luminal androgen receptor subtypes. To identify novel treatment strategies for TNBC patients, it is essential that we understand the unique and common molecular features of these TNBC subtypes.

Current treatment options for TNBC patients are restricted to chemotherapy; however, receptor tyrosine kinases (RTK) are promising druggable targets due to their high expression in TNBC. The epidermal growth factor receptor (EGFR) and MET receptor are highly expressed in multiple TNBC subtypes with EGFR overexpression in 54% of basal breast cancers (predominantly TNBC). Additionally, EGFR is a biomarker for identification of basal breast cancers [6–10]. Similarly, MET is associated with poor clinical outcome in breast cancer [11–15], and high MET expression correlates with TNBC [16, 17]. Previously, we demonstrated that the MET inhibitor cabozantinib inhibited TNBC growth, invasion, and metastasis [18]. Recently, we determined that combined MET and EGFR inhibition was highly effective at abrogating tumor growth in patient-derived TNBC tumorgrafts and significantly decreased the variability in treatment response compared to monotherapy with MET or EGFR inhibitors [19]. These results highlight that MET and/or EGFR inhibition may be a highly effective treatment strategy for TNBC patients. Metabolic alterations are now widely understood to support the cancer phenotype, and RTKs such at MET and EGFR have been implicated in driving some of these the metabolic alterations [20–25].

### Metabolic characteristics of TNBC

Particular metabolic characteristics of TNBC have been investigated, and overall TNBC cell models and patient samples are characterized by elevated glycolysis. Along these lines, a genome wide screen identified a small subset of metabolic genes, including core glycolytic and oxidative phosphorylation (OXPHOS) genes, whose suppression was lethal in a TNBC cell model [26]. Compared to ER+ breast cancer cell lines, MDA-MB-231 and MDA-MB-468 TNBC cell models are reported to harbor high glycolytic flux and low OXPHOS activity [27] and are more primed to switch to a glycolytic program in the context of limited oxygen than non-transformed cells [28]. In patient samples, high glucose transporter, GLUT1, expression is observed in TNBC compared to non-TNBC tumors [29]. GLUT1 may also enhance invasion by localizing to the invasive edge of in vivo tumor models [30]. Mechanistically, high MYC expression in TNBC cell models suppresses expression of the glycolytic inhibitor, thioredoxin-interacting protein, TXNIP, resulting in increased glycolytic flux [31]. In addition, a siRNA screen revealed that TNBC cell line models are dependent on elevated glycolysis through the LDHB (lactate dehydrogenase B) as opposed to their non-TNBC counterparts [32].

Recent evidence indicates that the metabolic characteristics of TNBC correlate with therapeutic response. The glycolytic potential of TNBC cells may be associated with chemotherapeutic resistance as exposing TNBC cell models to increasing concentrations of glucose increases proliferation and decreases the efficacy of metformin-induced apoptosis [33, 34]. Additionally, PKM2, a glycolytic enzyme associated with high tumoral glycolytic flux [35], may confer some resistance to doxorubicin treatment in vitro and in MDA-MB-231 orthotopic breast cancer models [36]. Other studies demonstrate that stimulation of mitochondrial activity and concurrent inhibition of mitochondrial respiratory complex I [37] or a combination of glycolytic and mitochondrial inhibitors [38] effectively kills TNBC cells and TNBC xenografts.

Collectively, the above studies demonstrate a clear role for altered metabolism supporting the aggressive TNBC phenotype. Much like the genetic and signaling heterogeneity found in cancers in general and TNBC in particular [39], metabolic heterogeneity also likely exists in TNBC patients [40, 41] and cell models [33, 42] and likely drives differential responses to therapeutics. Therefore, comprehensive and systematic investigations into the metabolism of TNBC and TNBC cell models are necessary in order to gain insight into the best therapeutic strategies. Some previous approaches referenced above undertook genome-wide siRNA screening approaches or utilized computational approaches [43]. In the present study, we provide detailed metabolic analyses of the commonly used MDA-MB-231, MDA-MB-468, Hs578T, and HCC70 TNBC cell line models, which represent the two major basal-like subtype and the mesenchymal subtypes [4]. We determined the basal and maximal metabolic rates, as well as the metabolic rates in response to EGFR and MET inhibitors in these TNBC lines. We also measure viability in response to chemical modulation of five metabolic pathways. Finally, we report the viability effects of suppressing each KEGG metabolic pathway in combination with EGFR (erlotinib) or MET (INC280) inhibition in these cell lines. Overall, these results provide a more thorough view of the metabolic landscape of TNBC and the effect of RTK inhibition on TNBC metabolism.

## Methods

### Cell culture

All cells were purchased from ATCC. MDA-MB-231, MDA-MB-468, Hs578T, and HCC70 cells were cultured in DMEM (ThermoFisher) supplemented with 10% fetal bovine serum. hTERT-HME1 cells were cultured in MEBM (Lonza) supplemented with hEGF, insulin, hydrocortisone, and BPE (Lonza).

### Metabolomics profiling

For metabolite quantification, cells were seeded in triplicate (*n* = 3) in 6-well plates with DMEM supplemented with 10% FBS. After 24 h, the media was removed and replaced with fresh media. Upon reaching 70% confluency, cells were washed twice with phosphate buffered saline (PBS, 46-013-CM, Corning) and lifted from culture wells using 0.25% Trypsin/2.21 mM EDTA (25-053-CI, Corning). Cells were then washed with PBS containing 10% FBS followed by 0.9% NaCl (Sigma, S9888). Cell pellets frozen in liquid nitrogen before storage at 193 K.

Frozen cell pellets were thawed on ice for 10 min before addition of 1 mL cold extraction solvent containing acetonitrile/isopropanol/water (3:3:2) at 253 K. Samples were then vortexed (15 s × 5) and frozen on dry ice for 20 mins and the freeze/thaw/vortex cycle repeated twice. Samples were dried via vacuum centrifugal evaporation and stored at −80 °C before analysis.

Dried samples were derivatized first by addition of 10 μL of MOX Reagent (20 mg/mL methoxyamine-hydrochloride in dry pyridine (TS-45950, Thermo Fisher Scientific) followed by 90-min incubation in a digital heating shaking drybath at 303 K and 1100 rpm. Next, 90μL N-Methyl-N-(trimethylsilyl)trifluoroacetamide (MSTFA, Sigma 394,866) was added and samples were incubated at 310 K and 1000 rpm for 30mins before centrifugation for 5 min at 14,000 rpm/277 K. The supernatant was transferred to an auto sampler vial for gas chromatography-mass spectrometry (GC-MS) analysis.

Derivatized samples were analyzed on a Triple Quadrupole GC-MS (TSQ8000, Thermo Fisher Scientific) equipped with a TG-5MS (30 m × 0.25 mm i.d. × 0.25 μm, 26098-1420, Thermo Fisher Scientific) capillary column and run under electron ionization at 70 eV. The GC was programed with an injection temperature of 523 K and splitless injection volume of 1 μl. The GC oven temperature program started at 232 K for 1 min, rising to 523 K at 10 K/min with a final hold at this temperature for 6 min. The GC flow rate with helium carrier gas was 1.2 mL/min. The transfer line temperature was set at 563 K and ion source temperature at 568 K. A range of 50–600 m/z was scanned with a scan time of 0.25 s.

Metabolites were identified using TraceFinder software v 3.3 (Thermo Fisher Scientific) based on in-house libraries of metabolite retention time and fragmentation patterns. Identified metabolites were quantified using the total ion count peak area for specific mass ions, and standard curves generated from reference standards run in parallel. The mean, standard deviation, and 95% confidence interval were calculated for each cell line and treatment condition. ANOVA with student’s *t* test was used to compare treatment conditions within each cell line.

### Metabolic flux analysis

For all metabolic flux analyses, a Seahorse 96 XFe was used. Twenty-four hours prior to metabolic flux analyses, cells were cultured in identical media (10 mM glucose, 2 mM glutamine, 1 mM pyruvate). Cells were plated at a density of 40,000 cells per well in a Seahorse 96-well assay plate 16 h prior to analysis. For basal and maximal metabolic profiles, four independent experiments were performed, each with three biological replicates and five technical replicates. For basal metabolic profiles in the context of RTK inhibitor treatment, three biological replicates each with five technical replicates were performed, and cells were treated with 10 μM erlotinib or INC280/capmatinib (Selleck Chemicals) for 18 h prior to metabolic rate analysis. After metabolic rate analyses, extracellular acidification rate (ECAR) and oxygen consumption rate (OCR) measurements were normalized to CyQUANT (Invitrogen) measurements cell count measurements in each well. For basal rate measurements, ECAR and OCR measurements were spaced 6 min apart. For maximal rate measurements, basal rates were measured twice at an interval of 6–7 min, followed by carbonyl cyanide-p-trifluormethoxyphenylhydrazone (FCCP) (1 μM final concentration) injection, mixture, and measurement 6–7 min later, followed another measurement 6–7 min later, followed by 2-deoxyglucose (2-DG, 100 mM final concentration) or rotenone + antimycin (1 μM each final concentration) injection, mixture, and measurement 6–7 min later, followed by a final measurement 6–7 min later. Maximal rate data are representative experiments displayed as averages of three biological replicates with error bars representing standard deviation.

### Cell viability in response to metabolic modulators

Cells were plated at a density of 2500 cells per well in 96-well plates in growth media. Cells were treated with vehicle or the following concentrations of chemicals: 25 mM 2-DG, 200 μM 6-aminonicotanimide (6-AN), 1 μM rotenone, 10 mM metformin, and 1 mM 5-Aminoimidazole-4-carboxamide ribonucleotide (AICAR). After 48 h of treatment, viability was measured by CellTiter-Glo (Promega). Two independent experiments, each containing six biological replicates, were performed. Data are from one representative experiment and provided as averages with error bars representing standard deviation.

### siRNA screen

#### Screen design

All small interfering RNAs (siRNAs) were from Qiagen (Additional file 1: Table S1) and were transfected into cells with siLentFect (BioRad, 1 μl per ml, for transfection efficiency for each cell line, see Additional file 2: Figure S1A). Rate-limiting enzymes were collated through KEGG annotation (http://www.genome.jp/kegg/), the Rate-Limiting Enzyme Regulation Database (http://rle.cbi.pku.edu.cn/home.cgi, [44]), and literature searches and categorized according to KEGG. Genes and metabolic categories and pathways are provided in Additional file 3: Table S2 according these KEGG-based annotations. For the siRNA screen, cells were transfected with control (non-targeting) siRNAs or siRNAs targeting the above-described rate-limiting enzymes, then treated with either DMSO, INC280, or erlotinib (Additional file 2: Figure S1A). Cells were plated in 96-well assay plates at 2500 cells per well. Sixteen hours later, cells were transfected with a pool of two siRNAs per gene. Twenty-four hours post-transfection, fresh media was added containing 10 μM INC280, 10 μM erlotinib, or 0.1% DMSO (the final DMSO concentration in wells containing INC280 or erlotinib). The screen was carried out in duplicate for each siRNA and each condition (DMSO, INC280, or erlotinib) in each cell line. Seventy-two hours post-transfection (48-h post-drug treatment), cell viability was assessed by CellTiter-Glo (Promega).

#### Screen analysis

The siRNA screen was performed in duplicate, and sensitivity index (below) values were derived from replicate averages. Replicates resulting in a variance larger than 0.04 were not considered for further analysis. To determine which siRNAs resulted in the greatest loss of viability in combination with INC280 or erlotinib compared to DMSO, a variation on the sensitivity index (SI) equation developed by Hoffman and Gardner (1983) was used to estimate the effect of siRNA knockdown on drug sensitivity [45]. The SI value for each siRNA was calculated using the following equation:

$$ SI=\left(\frac{R_{\mathrm{c}}}{C_{\mathrm{c}}}\times \frac{C_{\mathrm{d}}}{C_{\mathrm{c}}}\right)-\left(\frac{R_{\mathrm{d}}}{C_{\mathrm{c}}}\right). $$

In this equation, *R*_c_ is the average viability in drug-untreated (DMSO) wells transfected with siRNA targeting rate-limiting enzymes, *R*_d_ is the average viability in drug-treated wells (INC280 or erlotinib) with siRNA targeting rate-limiting enzymes, *C*_c_ is the average viability in drug-untreated (DMSO) wells with control (non-targeting) siRNA, and *C*_d_ is the average viability in drug-treated (INC280 or erlotinib) wells with control (non-targeting) siRNA [46]. The SI ranges from −1 to 1, with negative values indicating an antagonistic effect on drug performance and positive values indicating a sensitizing effect. This is accomplished by comparing the predicted effect of drug and siRNA exposure (*R*_c_/*C*_c_ × *C*_d_/*C*_c_) to the observed effect of combined exposure (*R*_d_/*C*_c_). Although the SI allows for rapid analysis of siRNA screening data that surpasses the power observed in simple fold-change analysis, it does not allow for the calculation of a *p* value, as it does not consider probability distribution [46, 47]. As a result, the top 10% of sensitizing siRNAs was used in metabolic pathway analysis. These resulting genes were grouped into KEGG-annotated Metabolic Categories for each drug treatment in each cell line and into KEGG-annotated Metabolic Pathways for each drug treatment.

## Results

### Metabolomics profiles of TNBC cell lines

To understand the diversity of metabolic activity in TNBC, we examined multiple TNBC cell lines that are representative of several TNBC subtypes identified by Lehmann et al. [4]. These cell lines correspond to the two major basal-like subtypes and a mesenchymal-like subtype. This included Hs578t (mesenchymal stem-like; basal B), MDA-MB-231 (mesenchymal stem-like; basal B), MDA-MB-468 (basal-like 1; basal A), and HCC-70 (basal-like 2, basal A) cells (Additional file 2: Figure S1B). To produce initial metabolic profiles of TNBC, we measured the basal glycolytic and mitochondrial oxidative metabolism rates in four TNBC cell models (MDA-MB-231, MDA-MB-468, HS578t, HCC70) and one immortalized, non-transformed mammary gland epithelial cell model (hTERT-HME1) (Additional file 2: Figure S1C).

**Fig. 1:**
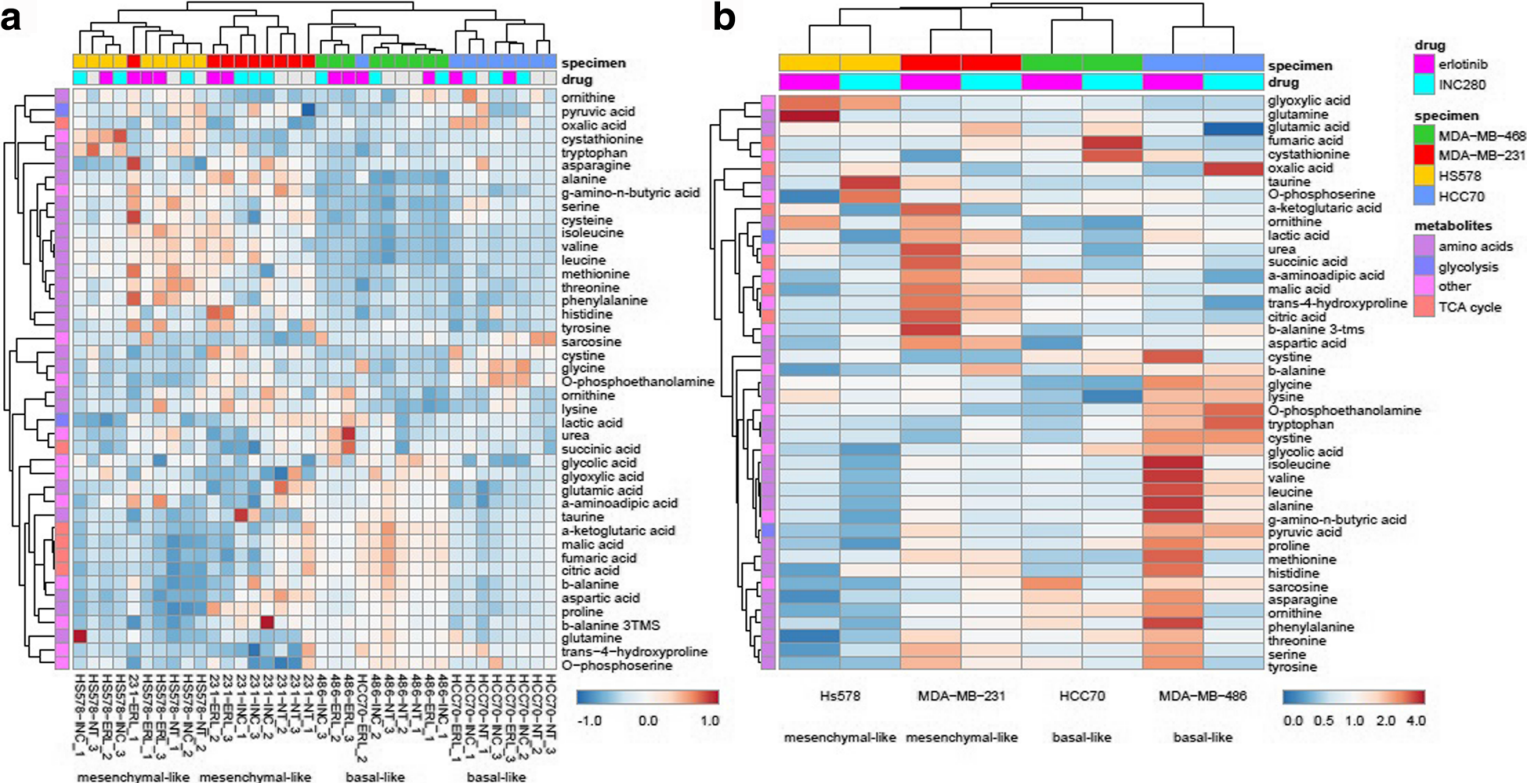
Metabolomics profiling of TNBC cell lines. Hierarchical clustering of metabolic profiles of TNBC cell lines reveals molecular heterogeneity between subtypes. **a** Pool size measurements show common clusters of low TCA cycle and elevated amino acid metabolites of mesenchymal-like subtype cell lines MDA-MB-231 and Hs578 vs basal-like subtypes HCC70 and MDA-MB-456. **b** Clustering of drug responses of TNBC cell lines (average ratios of metabolite concentrations in conditions INC280/vehicle and erlotinib/vehicle are plotted for each set of biological triplicates). Drug perturbations of reduced amino acid pool sizes show similar response of reduced amino acid pool sizes upon receptor tyrosine kinase inhibitor treatment of mesenchymal-like subtype MDA-MB-231 and Hs578 cell lines. INC280/capmatinib was used to inhibit proto-oncogene MET receptor tyrosine kinase, and erlotinib was used to inhibit receptor tyrosine kinase and growth factor receptor EGFR in TNBC cell lines

We profiled pool sizes of 43 central carbon metabolites of subconfluent TNBC cell lines in exponential growth phase. In addition, we quantified pool size changes following treatment with small molecule inhibitors of the RTKs MET and EGFR. Both MET and EGFR were prominently expressed in the assayed cell lines. Hierarchical clustering of metabolic profiles of TNBC cell lines reveals molecular heterogeneity between the TNBC mesenchymal-like and basal-like subtypes (Fig. 1). Pool size measurements showed common clusters of low TCA cycle and elevated amino acid metabolites of mesenchymal-like MDA-MB-231 and Hs578 which were distinct from the basal-like MDA-MB-468 and HCC70 cell lines (Fig. 1a, Additional file 4: Table S3). Drug perturbations of amino acid pool sizes demonstrated similar response of mesenchymal-like subtype MDA-MB-231 and Hs578 cell lines to both, INC280 or erlotinib, treatment (Fig. 1b). Clusters of each subtype and cell line were well separated by metabolic profiles and drug responses showing that each subtypes had major similarities but each breast cancer cell line also had distinct components. The TCA cycle organic acid α-ketoglutaric acid is significantly reduced upon INC280 treatment with *p* values below 0.05 for all tested TNBC cell lines. Similarly, TCA cycle and central carbon metabolites aspartic acid, fumaric acid, and malic acid are significantly reduced upon erlotinib treatment with *p* values below 0.05 for all cell lines. In addition, the MDA-MB-231 cell lines show significant perturbation of amino acid metabolism for both inhibitors. Interesting, the MDA-MB-231 cell line stands out for its strong metabolic perturbation affecting TCA cycle metabolites, many amino and keto acids (Fig. 1b).

### TNBC basal metabolic profiles

**Fig. 2:**
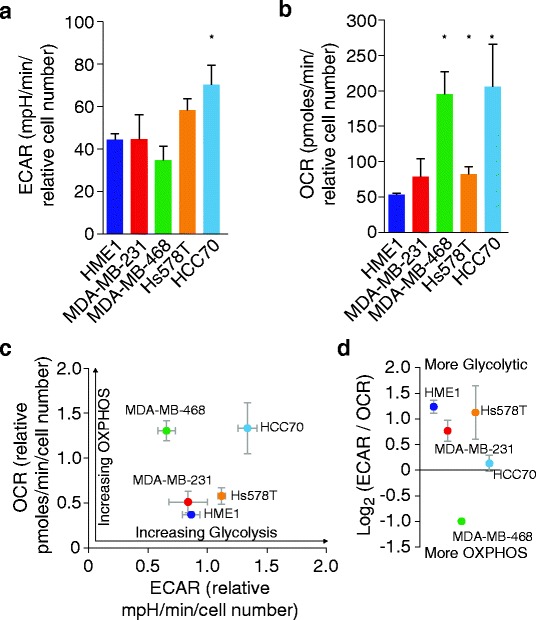
TNBC basal metabolic profiles. **a** Cells were maintained in uniform media for 24 h prior to measuring ECAR. ECAR values were normalized to total cell numbers for each cell line in the ECAR assay. Data are ECAR averages from four experiments, each with five biological replicates. Error bars represent SEM. Asterisks indicate significance compared to HME1 cell values (*p* ≤ 0.05). **b** Cells were maintained in uniform media for 24 h prior to measuring OCR. OCR values were normalized to total cell numbers for each cell line in the OCR assay. Data are OCR averages from four experiments, each with three biological replicates. Error bars represent SEM. Asterisks indicate significance compared to HME1 cell values (*p* ≤ 0.05). **c** Relative ECAR and OCR data from **a** and **b** were plotted simultaneously to reveal overall relative basal metabolic profiles for each cell model. **d** The ECAR/OCR ratio of the data in **d** was log2 transformed to provide an index of each cell model’s comparative utilization of glycolytic and oxidative metabolism

MDA-MB-231 and MDA-MB-468 cells exhibited similar glycolytic rates (extracellular acidification rate, ECAR) compared to HME1 cells, while HS578t and HCC70 cells displayed approximately 1.5 and two times the glycolytic rate of HME1 cells, respectively (Fig. 2a, c). MDA-MB-231 and HS578t cells exhibited slightly elevated oxygen consumption rates (OCR) compared to HME1 cells, whereas MDA-MB-468 and HCC70 displayed approximately four times the oxygen consumption rate of HME1 cells (Fig. 2b, c). Determining each cell lines’ relative ECAR/OCR ratio provides a relative index of which metabolic program each cell line utilizes more in the basal state (Fig. 2d). HME1 cells utilize relatively more basal glycolytic than oxidative metabolism, as do Hs578T and MDA-MB-231 cells. HCC70 cells utilize relatively similar basal glycolytic and oxygen metabolism, while MDA-MB-468 cells utilize relatively more oxygen metabolism than glycolytic metabolism (Fig. 2d). Together, the analyses of basal metabolic rates indicate that HME1, MDA-MB231, and Hs578T are all more poised to rely on glycolytic metabolism, while MDA-MB-468 cells are more poised to rely on oxidative metabolism in the basal state. Interestingly, in the basal state, HCC70 cells exhibited the greatest glycolytic and oxidative metabolism rates (Fig. 2a–c), but also exhibited the most balance between these rates (Fig. 2d). Understanding basal metabolic rates and the relative metabolic index may provide insight into which metabolic program specific cancers or cancer cell models may be especially sensitive (Fig. 4).

### TNBC maximal metabolic profiles

**Fig. 3:**
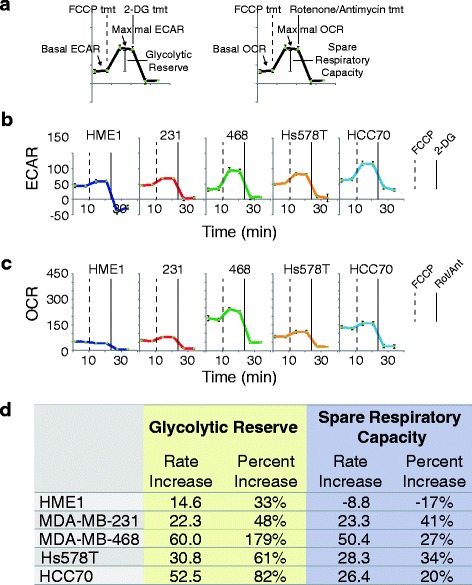
TNBC maximal metabolic profiles. **a** Schematic indicating derivation of maximal metabolic rates, glycolytic reserve, and spare respiratory capacity from the experiments in (**a**) and (**b**). **b** Cells were maintained in uniform media prior to measuring ECAR. ECAR was measured twice in the basal state, and then twice again following each metabolic inhibitor treatment (1 μM FCCP, 50 mM 2-deoxyglucose) at 7 minute intervals. ECAR values were normalized to a measurement of total cell numbers for each cell line in the ECAR assay. Data are ECAR averages from one representative experiment, with error bars representing SD. **c** Cells were maintained in uniform media prior to measuring OCR. OCR was measured twice in the basal state, and then twice again following each metabolic inhibitor treatment (1 μM FCCP, 1 μM rotenone + 1 μM antimycin) at 7 minute intervals. OCR values were normalized to a measurement of total cell numbers for each cell line in the OCR assay. Data are OCR averages from one representative experiment, with error bars representing SD. **d** Glycolytic reserve (derived from **b**) and spare respiratory capacity (derived from **c**) calculations. Data are expressed as actual rate unit increase and percent increase over basal rates. Rate increases were calculated by subtracting the basal rate values from the maximal rate values. Percent increases were calculated by dividing the rate increase values by the basal values

While basal metabolic rates (Fig. 2) are informative, most cells harbor the ability to alter one metabolic program in order to compensate when another metabolic program is perturbed. Therefore, inhibition of one metabolic program (e.g., glycolysis) allows measurement of the maximal capability of the other metabolic program (e.g., oxidative metabolism) (Fig. 3a). To determine the maximal glycolytic and oxygen consumption metabolic capabilities (Additional file 5: Figure S2A, B) in these TNBC cell models, we measured respiration arrest-induced maximal glycolytic rates and depolarization-induced maximal OXPHOS rates (Fig. 3b, c). The MDA-MB-231 cell line and the non-transformed mammary gland epithelial cell model, HME1, exhibited the least metabolic flexibility, as demonstrated by only moderate adjustments in ECAR and OCR (changes in values post-FCCP addition, Fig. 3b) and subsequent calculated glycolytic reserve and spare respiratory capacity rates (Fig. 3c). Interestingly, these two cell lines also displayed very modest basal metabolic rates (Fig. 2). Hs578T maximal ECAR and OCR were moderately elevated above basal rates, above those of HME1 and MDA-MB-231 but below MDA-MB-468 and HCC70. Both MDA-MB-468 and HCC70 maximal ECAR were nearly double basal rates, with HCC70 displaying the greatest glycolytic capacity. MDA-MB-468 and HCC70 maximal OCR were moderately elevated above basal rates, and MDA-MB-468 displayed the greatest capacity for oxidative metabolism. From these measurements, glycolytic reserve and spare respiratory capacity can be calculated (Fig. 3a). While all cell models displayed some glycolytic reserve, HCC70 and MDA-MB-468 cells exhibited the greatest glycolytic reserves (Fig. 3d). MDA-MB-468 also exhibited the greatest spare respiratory capacity (Fig. 3d). These data indicate that each of the TNBC cells possesses a measure of metabolic flexibility as defined by their abilities to increase ECAR or OCR when the one program is perturbed, with MDA-MB-468 cells exhibiting the greatest metabolic flexibility.

### TNBC response to metabolic modulators

**Fig. 4:**
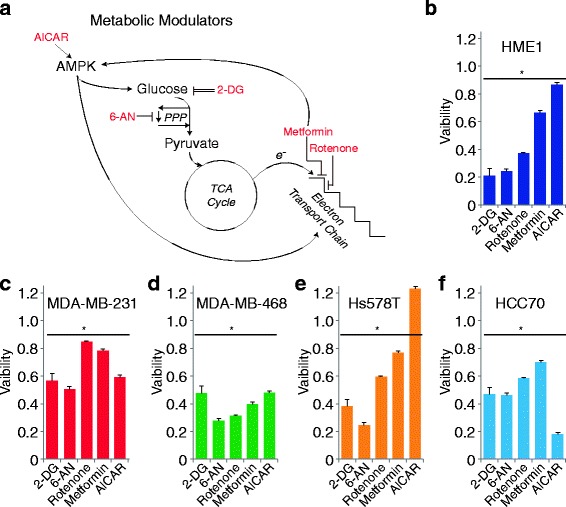
TNBC response to metabolic modulators. **a** Schematic of metabolic pathways and the points at which chemical inhibitors or activators act. **b**–**d** Viability measurements of cells following 48 h of treatment with indicated chemicals: 25 mM 2-DG, 200 μM 6-AN, 1 μM rotenone, 10 mM metformin, and 1 mM AICAR. Data are viability averages from one representative experiment with error bars representing SD. Asterisks represent significance compared to vehicle control (*p* ≤ 0.01)

To further characterize the metabolic profiles of these cell models, we assessed the effects on viability following treatment with metabolic modulators at concentrations commonly utilized in published literature (Fig. 4). These experiments evaluated the effects of the 5′ adenosine monophosphate-activated protein kinase (AMPK) activator [5-aminoimidazole-4-carboxamide-1β riboside (AICAR)] [48], the glycolytic inhibitor 2-deoxy-glucose (2DG) [49], the pentose phosphate inhibitor 6-amino-nicotinamide (6-AN) [50], the mitochondrial complex I inhibitor rotenone [51], and the AMPK activator/Complex I inhibitor metformin [52, 53] (Fig. 4a). Similar to the metabolic rate investigations above, the TNBC models exhibited heterogeneous responses to these treatments; however, from these results, some interesting patterns were observed (Fig. 4b–f). Each TNBC model exhibited an approximately 40–60% loss of viability in response to the glycolytic inhibitor 2-DG. Interestingly, HME1 cells, which exhibited the greatest bias towards basal utilization of glycolytic metabolism (Fig. 2e), were most affected by 2-DG treatment, as well as the pentose phosphate inhibitor 6-AN (Fig. 4b). Conversely, MDA-MB-468 cells, which exhibited the greatest bias towards basal utilization of oxidative metabolism (Fig. 2e), were most affected by the electron transport chain inhibitor, rotenone (Fig. 4d).

Also of note, Hs578T cells exhibited enhanced proliferation in response to AICAR while other cell lines exhibited variable decreases in viability compared to control. While AICAR and metformin are both commonly used as AMPK activators, they activate AMPK via disparate mechanisms. However, metformin and rotenone both perturb oxidative phosphorylation through respiratory chain complex I (RCI) inhibition. Our results reveal more similar viability effects between metformin and rotenone (common RCI inhibitors) than between metformin and AICAR (common AMPK activators) (Fig. 4b–f).

### Comprehensive analysis of rate-limiting enzymes and RTK inhibition

**Fig. 5:**
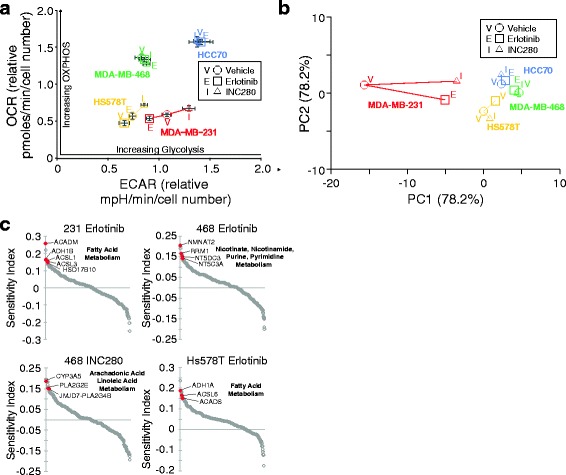
RTK-dependent TNBC sensitization to metabolic pathway perturbation. **a** Metabolic rate response to RTK inhibitors. 40,000 cells per well were plated in Seahorse 96-well assay plates. Cells were treated for 24 h with the DMSO, erlotinib, or INC280, and then ECAR and OCR were measured as described in Methods. **b** Principle component analysis of drug responses. Mesenchymal-like subtype MDA-MB-231 and Hs578 cell lines show largest perturbations. **c** RNAi screen results for selected TKI treatments which induce sensitivities to knockdown of common metabolic pathway genes

EGFR and MET receptors are known to drive tumorigenic progression, and RTKs are known to regulate metabolic signaling pathways [54]. To investigate the effects of EGFR and MET inhibition on TNBC metabolism, we measured ECAR and OCR in TNBC cells treated with the EGFR inhibitor erlotinib and the MET inhibitor INC280 (capmatinib). As in the above analyses, the TNBC cell models displayed heterogeneous responses to the tyrosine kinase inhibitors (TKIs). We observed that MET and EGFR inhibition had little effect on the glycolytic and oxidative metabolism rates of basal A/B subtype MDA-MB-468 or HCC70 cell lines (Fig. 5a). In contrast, in both mesenchymal-like MDA-MB-231 and Hs578T cell lines, both MET and EGFR inhibition strongly perturbed both glycolysis and oxidative metabolism (Fig. 5a). Importantly, comparing these data to a principal component analysis of our metabolomics data revealed that metabolic changes of mesenchymal-like MDA-MB-231 and Hs578T cell lines upon drug treatment recapitulated the observed changes of metabolic fluxes (Fig. 5b). Both cell lines show perturbation of the top two principal components (reflecting 84.9% of the data) in the same direction and magnitude. In contrast, major principle components of metabolic perturbations do not change for basal-like subtype MDA-MB-468 or HCC70. Despite the metabolic responses of MDA-MB-231 and Hs578T cell lines to INC280 vs erlotibib based on amino acid and TCA cycle metabolism of mesenchymal-like cell lines are in agreement, glycolytic rates show differential perturbation. Among the assessed TNBC cell lines, mesenchymal-like subtypes showed strong, consistent perturbations, despite underlying heterogeneity of breast cancer subtypes.

To more comprehensively investigate the metabolic consequences of EGFR and MET inhibition in TNBC models, we performed a siRNA screen in each cell line targeting all 323 rate-limiting enzymes in the human KEGG metabolic pathways and Rate Limiting Enzyme Regulation databases. This compliment of enzymes was divided into 11 major metabolic categories representing 89 metabolic pathways (Additional file 3: Table S2). The siRNA screen was performed in duplicate in each cell line in cells treated with vehicle (DMSO), INC280, or erlotinib. A sensitivity index was applied to viability measurements to determine the effect of siRNA knockdown on drug sensitivity, and therefore identifies genes whose knockdown preferentially reduces viability in TNBC cells treated with a TKI vs vehicle alone (see Methods, Additional file 6: Table S4). Eight of the 11 major metabolic categories were represented in the sensitivity index to varying levels for each TNBC model and each TKI (Additional file 7: Figure S3, see also Additional file 6: Table S4, Additional file 8: Table S5). Notably, lipid metabolism was prominent (represented > 15% of hits) in each TNBC model treated with INC280 or erlotinib, while amino acid metabolism was also prominent in each TNBC model treated with INC280. Evaluating individual significant rate-limiting enzymes in each group revealed potential sensitivities associated with specific TKI for some TNBC subtypes (Fig. 5c). MDA-MB-231 and Hs578T cells treated with erlotinib were sensitive to knockdown of fatty acid genes, while MDA-MB-468 cells treated with the same TKI were sensitive to knockdown of specific nucleotide metabolism pathways. INC280 sensitized MDA-MB-468 cells to knockdown of arachidonic and linoleic acid metabolism rate limiting enzymes. Interestingly, a broader analysis of metabolic pathways across cell TNBC subtypes in response to TKI treatments identified additional sensitivities. The top ten metabolic pathways identified by the sensitivity index displayed significant overlap between the TKI treatments (Additional file 7: Figure S3, see also Additional file 8: Table S5). Additionally, within this set of common pathways, three pathways which are engaged to counteract oxidative stress were enriched in the screen results (Glutathione metabolism, cytochrome P450 metabolism, and non-P450 drug metabolism pathways). These results suggest that even with the molecular heterogeneity that is present in TNBC, there are common metabolic programs that can be targeted in TNBC subtypes. Taken together, these data shows that targeting pathways such as fatty acid metabolism, pyrimidine metabolism, or oxidative stress relief pathways in combination with MET or EGFR inhibition may represent an effective therapeutic strategy.

## Discussion

In this study, we characterized the metabolic heterogeneity of TNBC and identified a core set of metabolic pathways that are common among the TNBC subtypes, yet observed diverse metabolic profiles among TNBC cell lines. Genetic and signaling heterogeneity is observed in most solid cancers, and studies have indicated that there is a particularly high level of genomic heterogeneity among TNBC patients [39]. These observations of heterogeneity have been borne out in metabolic analyses of TNBC as well. Analyses of TNBC patient tissues have demonstrated heterogeneity of glycolytic and mitochondrial protein expression [40, 41, 55]. TNBC cell lines have also exhibited heterogeneity with respect to glutamine metabolism [42] and response to the metabolic modulator, metformin [33, 34]. Therefore, the heterogeneity evident between TNBC cell lines in our detailed metabolic characterization extends these previous findings. Metabolic pool sizes and drug responses revealed common patterns between TNBC subtypes but also highlighted cell line-specific responses (Fig. 1). Importantly, drug responses of metabolic rates and principle metabolic components identified a theme of metabolic capacity and adaptability as major difference of mesenchymal-like vs basal-like subtypes (Fig. 5) [56]. Basal-like cell lines are metabolically most active with the highest OCR and ECAR (Fig. 2c), resulting in low, depleted metabolic pool sizes (Fig. 1a). Mesenchymal-like cell lines have significantly lower OXPHOS allowing them to modulate and adaptively respond to the drug challenges (Fig. 5a, b). The HCC70 cell is an example of extremely high OCR and ECAR that allows for minimal adjustment to drug challenges. The unique metabolic profiles (Figs. 1, 2, and 3) response to chemical modulators (Fig. 4) and sensitivities to combined RTK, and metabolic pathway inhibition (Fig. 5) provides platforms which can help place the responses of these TNBC cell line models in previous and future studies into a broader metabolic context. For example, previous work demonstrated that MDA-MB-468 cells are more sensitive to metformin than MDA-MB-231 cells. Here, we provide a potential basis for that observation as we found MDA-MB-468 cells to be more poised to rely on OXPHOS than MDA-MB-231 cells (Fig. 2d). Therefore, this comprehensive metabolic analysis provides a platform in which to identify therapeutic sensitivities within the TNBC metabolic landscape.

In the TNBC cell models that we evaluated, an index mapping, the relative affinities for basal metabolic rates (Fig. 2d), appeared more useful for predicting response to chemical modulators (Fig. 4) than did an analysis of maximal metabolic rates (Fig. 3). Cells which displayed relatively higher OXPHOS rates than glycolytic rates (MDA-MB-468) were the most sensitive to rotenone treatment. On the other hand, cells which displayed relatively higher glycolytic rates than OXHPOS rates (HME1, Hs578T) were the most sensitive to 2-DG and 6-AN treatment. Therefore, although a cell may harbor the ability to greatly increase glycolytic rate when OXPHOS is disrupted (MDA-MB-468, Fig. 3a, d), its higher basal OXPHOS rate may reflect an absolute requirement for high, sustained oxidative metabolism. Therefore, it is possible that cells which display the ability to upregulate alternative metabolic pathways in response to metabolic insults still remain dependent on the metabolic pathways which they preferentially utilize in the basal state.

In addition to glycolytic and mitochondrial oxidative metabolism alterations, TNBC patient samples display evidence of altered glutamine metabolism compared to HER2 positive cancers [57]. TNBC cancer cell line models enhance glutamine uptake and metabolism, which are associated with epigenetic changes favoring expression of pro-tumorigenic genes [58, 59]. Other studies provide evidence of altered amino acid metabolism in TNBC. MDA-MB-231 and MDA-MB-468 cells exhibit elevated serine metabolism protein expression compared to HER2 positive cell lines, an observation that is shared in patient samples [60, 61]. Metabolomics analyses of patient samples identified lower amino acid levels in TNBC patients compared to healthy controls [62]. A folate metabolism enzyme may also serve as a biomarker for TNBC in distinct ethnic populations [63]. Finally, altered lipid metabolism appears to play a part in TNBC. Patient TNBC and non-TNBC tissues can be discriminated based on markers of lipid metabolism [64, 65], and MDA-MB-231 and MDA-MB-468 cells are effectively induced to undergo apoptosis upon suppressing expression of the lipogenic transcription factors, SREBP1/2 [66]. Recent metabolomics analyses identified metabolites associated with the Warburg effect, and the oxidative stress response, and specific metabolite signatures associated with different driver mutations [67]. Metabolomics have also identified potential global differences in breast cancer-associated metabolites between patients of different races [61], and miRNA-associated thiamine homeostasis specific to TNBC patient samples [68].

RTKs are promising drug targets due to their high expression in TNBC. The success of trastuzumab in HER2^+^ breast cancer underscores the potential of targeting tyrosine kinases yet, in spite of this promising start, monotherapy with tyrosine kinase inhibitors (TKIs) has had limited success in the clinic. In this study, we evaluated the effects of RTK inhibition on metabolic pathways in TNBC. This analysis highlighted the unique metabolic dependencies in our TNBC models, but also revealed reveal a core set of metabolic pathways that are universally affected by TKI treatment. Collectively, TNBC cells were commonly sensitized to inhibition of redox homeostasis, fatty acid metabolism, and nucleotide metabolism by erlotinib and INC280 treatment. The metabolomics results provide a mechanistic basis for the lipid metabolism sensitivity identified in the RLE siRNA screen. Flux analyses of multiple cancers demonstrate that altering TCA cycle flux significantly impinges upon lipid metabolism [69]. Therefore, the sensitivities to lipid metabolism RLE knockdown could be predicted by the concurrent changes in amino acid metabolites. Glutathione metabolism, cytochrome P450 metabolism, and non-P450 drug metabolism pathways, each of which ameliorate oxidative stress, were enriched in the siRNA screen, highlighting the importance of redox homeostasis in this context. Clinically, redox pathways have been shown to be upregulated in TNBC vs ER+ tumors [70] and heightened glycolytic metabolism may be regulated in part by oxidative stress in TNBC cells [71]. Our data provide additional impetus for co-targeting these metabolic and kinase pathways in TNBC patients [72]. siRNAs targeting fatty acid metabolism and specifically arachidonic acid metabolism genes were also common hits in the screen. Expression of fatty acid metabolism enzymes have previously been associated with TNBC metastasis [64] and survival rates [65]. Arachidonic acid metabolism itself may also be linked to cytochrome P450 metabolism in breast cancers [72]. Interestingly, suppression of tryptophan metabolism enhances INC280 treatment (Additional file 7: Figure S3). A previous investigation of BT549 TNBC cells demonstrated a link between tryptophan metabolism-dependent kynurenine production and breast cancer cell anoikis resistance, particularly in ER negative cell [73]. Because the data in Fig. 3d show that MDA-MB-468 cells have very little relative glycolytic activity while maintaining high OXPHOS activity, it would be reasonable to expect less effects from suppression of carbon metabolism RLEs. The data in Additional file 2: Figure S3 bear this out as carbon metabolism RLE knockdown has the least effect on MDA-MB-468 cells. A potential mechanism for this observation may be the KRAS mutational status of MDA-MB-468 cells which is not shared by the other TNBC lines under study. A previous study has demonstrated that some KRAS-driven cancers cells significantly upregulate OXPHOS metabolism [74]. Finally, MET or EGFR inhibition collectively sensitized TNBC cells to knockdown of pyrimidine and purine metabolism enzymes. Interestingly, a significant proportion of the siRNA hits driving enrichment of these metabolic pathways in our study are 5′-nucleotidases and nucleotide kinases. These results suggest that regulation of nucleotide phosphorylation plays an important role in determining sensitivity to RTK inhibitors in TNBC. Therefore, small molecules disrupting nucleotide phosphorylation dynamics may prove effective at enhancing RTK inhibition in TNBC.

## Conclusions

The findings in this study provide comprehensive information on the metabolic background of TNBC subtypes, their unique and common metabolic dependencies, and how they respond to metabolic insults. These results provide a valuable resource for investigators who utilize these TNBC cell lines. Additionally, our siRNA analysis establishes a comprehensive analysis of metabolic rate-limiting enzymes and identifies erlotinib- and INC280-sensitized pathways. Overall, this comprehensive metabolic analysis demonstrates the metabolic heterogeneity within TNBC and identifies therapeutic sensitivities that may be exploited in treating TNBC patients.

## Additional files

Additional file 1: Table S1. Genes targeted and siRNA sequences used in siRNA screen. (XLSX 57 kb)

Additional file 2: Figure S1. RNAi screen, cell lines, and metabolic rates. (A) Schematic of RNAi screen (left) and transfection efficiency under screen conditions (right). (B) Characteristics of each cell line used in this study. (C) Schematic representing biological compartments and metabolic pathways assessed for metabolic rates. (PDF 957 kb)

Additional file 3: Table S2. Genes and metabolic categories and pathways used in siRNA screen. Rate-limiting enzymes were collated through KEGG annotation (http://www.genome.jp/kegg/), the Rate-Limiting Enzyme Regulation Database (http://rle.cbi.pku.edu.cn/home.cgi, [51]), and literature searches and categorized according to KEGG. (XLSX 25 kb)

Additional file 4: Table S3. Quantitation of metabolic pool sizes, ratios for cell lines, and drug responses with statistical values. Total ion count integrated over peak area for metabolite-specific mass ion validated by multiple precursor-product ion combinations. Mean, standard deviation, and ANOVA with student’s t test compares triple-negative breast cancer (TNBC) cell lines and treatment with INC280 or erlotinib vs control vehicle (DMSO). (XLSX 52 kb)

Additional file 5: Figure S2. OCR and ECAR measurement explanation. (A) i. Normally functioning cellular respiration utilizes electrons in the form of NADH and FADH2 to pass down the electron transport chain (ETC) gradient. These redox reactions in the ETC pump hydrogen ions from the mitochondrial matrix into the mitochondrial inner membrane space (IMS), providing an electrochemical gradient which in turn powers ATP synthase-dependent ATP production. ii. FCCP is a lipid-soluble ionophore that allows hydrogen ions to escape the IMS, functionally uncoupling the ETC from ATP synthase-mediated ATP production. iii. In order to restore FCCP-mediated depletion of ATP levels, glycolytic flux increases to maximum capacity. iv. In order to maintain a minimal hydrogen ion gradient in the IMS, mitochondrial complex IV activity increases to maximum capacity, thus inducing maximum oxygen consumption. (B) The order of metabolic rate measurements and metabolic toxin treatment for maximal rate measurements. i. Basal glycolytic rate. ii. Respiration arrest-induced maximal glycolytic rate. iii. Glycolytic arrest. iv. Basal OXPHOS rate. v. Depolarization-induced maximal OXPHOS rate. vi. OXPHOS arrest. (PDF 997 kb)

Additional file 6: Table S4. Drug response and sensitivity index for siRNA treatment of triple-negative breast cancer (TNBC) cell lines.. (XLSX 128 kb)

Additional file 7: Figure S3. Sensitization to metabolic pathway perturbation. (A) Graphical representation of the proportion which each metabolic category (Table S2) was represented in the top 10% sensitivity index scores. (B) The combined top ten pathways as defined by high-scoring sensitivity index genes for each TKI (INC80 or erlotinib). (PDF 801 kb)

Additional file 8: Table S5. Top 10% scoring siRNAs in the siRNA screen for each condition. (XLSX 27 kb)

## Abbreviations

2-DG: 2-deoxyglucose
6-AN: 6-aminonicotanimide
AICAR: 5-Aminoimidazole-4-carboxamide ribonucleotide
AMPK: 5′ adenosine monophosphate-activated protein kinase
DMSO: Dimethyl sulfoxide
ECAR: Extracellular acidification rate
EGFR: Epidermal growth factor receptor
ER: Estrogen receptor, ESR1
HER2: Human epidermal growth factor receptor 2, NEU, ERBB2
INC280: Capmatinib, MET small molecule inhibitor
KEGG: Kyoto Encyclopedia of Genes and Genomes
MET: Hepatocyte growth factor receptor
OCR: Oxygen consumption rate
OXPHOS: Oxidative phosphorylation
PR: Progesterone receptor, PGR
RCI: Respiratory chain complex I
RTK: Receptor tyrosine kinase
SI: Sensitivity index
siRNA: Small interfering RNA
TKI: Tyrosine kinase inhibitor
TNBC: Triple negative breast cancer
