# Supplementary material for: Metabolic profiling of triple-negative breast cancer cells reveals metabolic vulnerabilities
Source: Cancer Metab. 2017 Aug 22;5:6. doi: 10.1186/s40170-017-0168-x (PMC5568171; doi:10.1186/s40170-017-0168-x)
Supplement: Supplementary file 2 — RNAi screen, cell lines, and metabolic rates. (A) Schematic of RNAi screen (left) and transfection efficiency under screen conditions (right). (B) Characteristics of each cell line used in this study. (C) Schematic representing biological compartments and metabolic pathways assessed for metabolic rates. (PDF 957 kb) [file 40170_2017_168_MOESM2_ESM.pdf]

Figure S1

A.

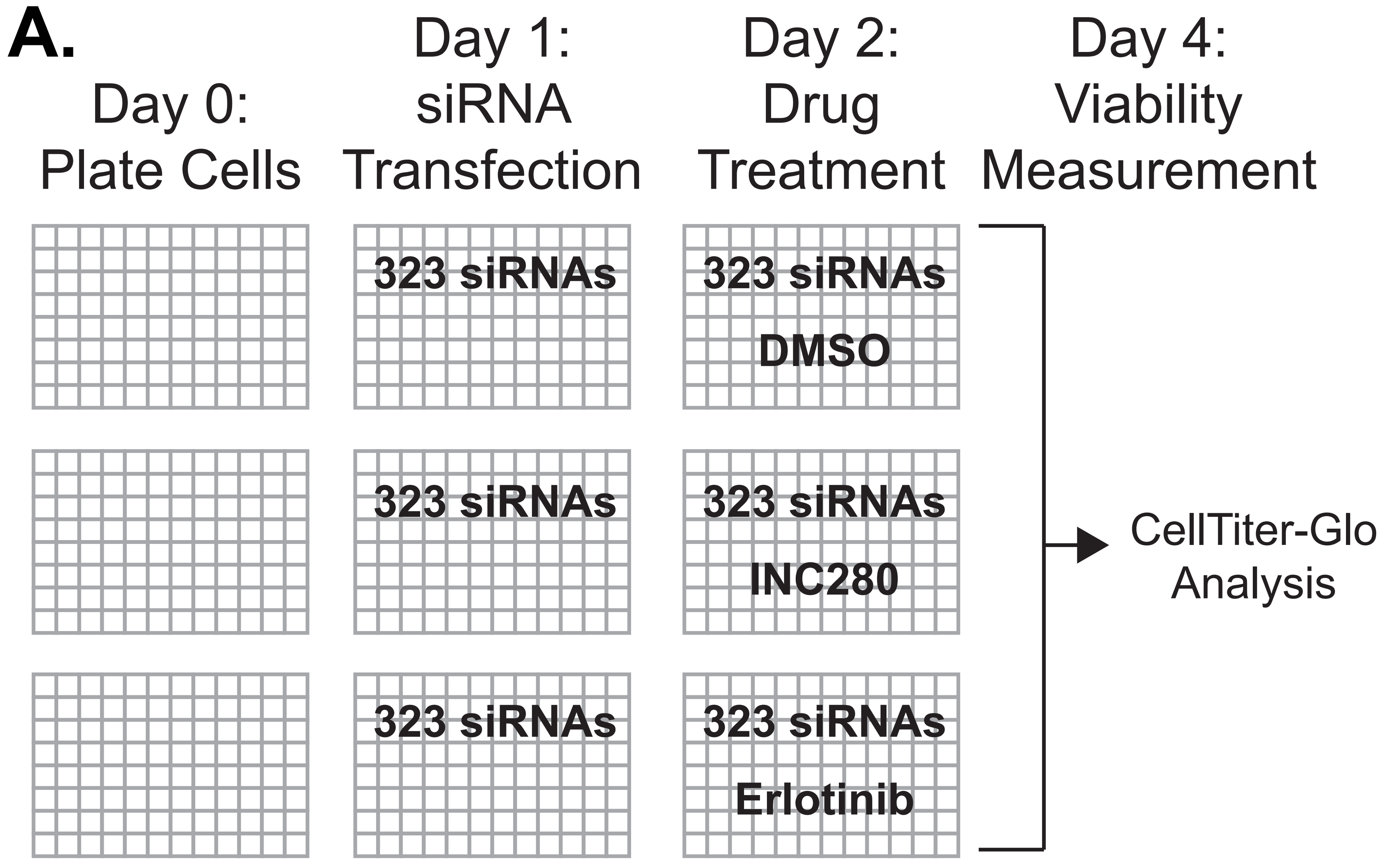

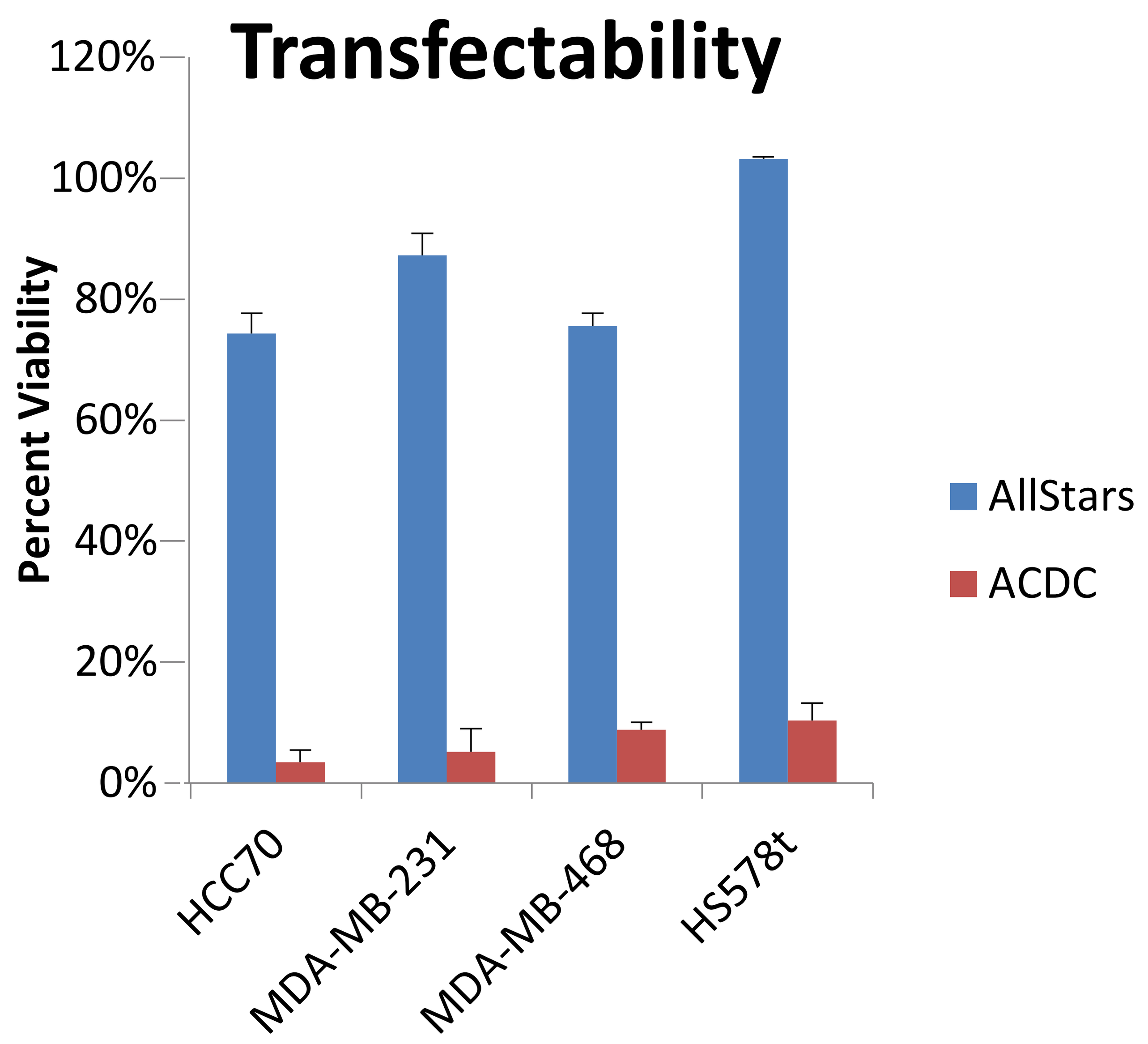

Analysis

$$\text{Sensitivity Index} = \left(\frac{\text{RLE siRNA + DMSO}}{\text{Control siRNA + DMSO}} \times \frac{\text{Control siRNA + Drug}}{\text{Control siRNA + DMSO}}\right) - \left(\frac{\text{RLE siRNA + Drug}}{\text{Control siRNA + DMSO}}\right)$$

C.

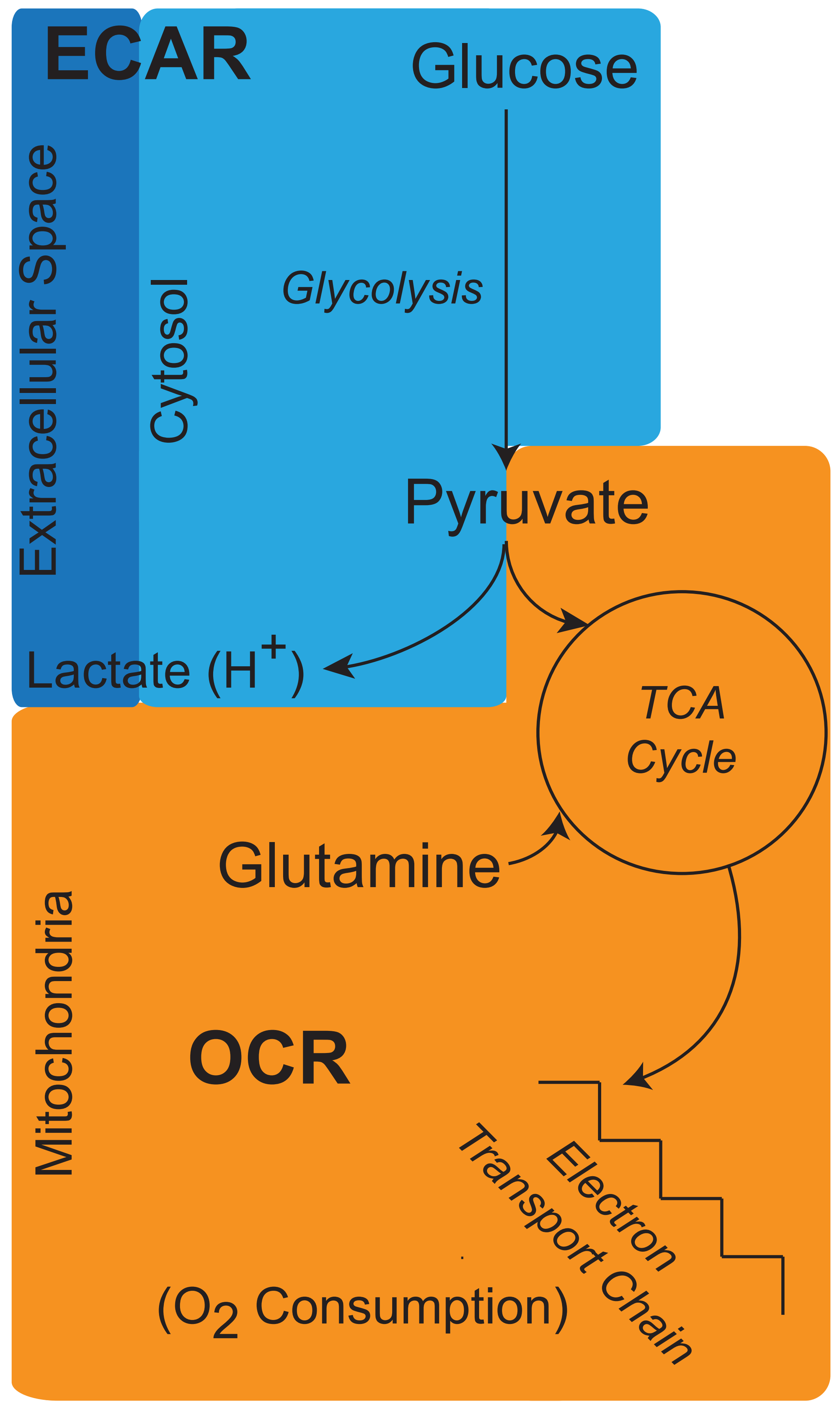

B.

Cell Line	TNBC Subtype	Source	Morphology	Mutations
hTERT-HME1	N/A	Immortalized Mammary Gland Epithelium	Epithelial	N/A
MDA-MB-231	Mesenchymal stem-like; Basal B	TNBC Metastatic	Epithelial	TP53; PDGFRA; NF2; BRAF
MDA-MB-468	Basal-like 1; Basal A	TNBC Metastatic	Epithelial	PTEN; ERBB2
Hs578T	Mesenchymal stem-like; Basal B	TNBC Metastatic	Epithelial	TP53; PIK3R1; NF1
HCC70	Basal-like 2; Basal A	TNBC Primary Ductal Carcinoma	Epithelial	TP53; RB1; PTEN; APC
