# Supplementary figures and images for: Metabolic profiling of triple-negative breast cancer cells reveals metabolic vulnerabilities
Source: Cancer Metab. 2017 Aug 22;5:6. doi: 10.1186/s40170-017-0168-x (PMC5568171; doi:10.1186/s40170-017-0168-x)

Figure S2

**A.**

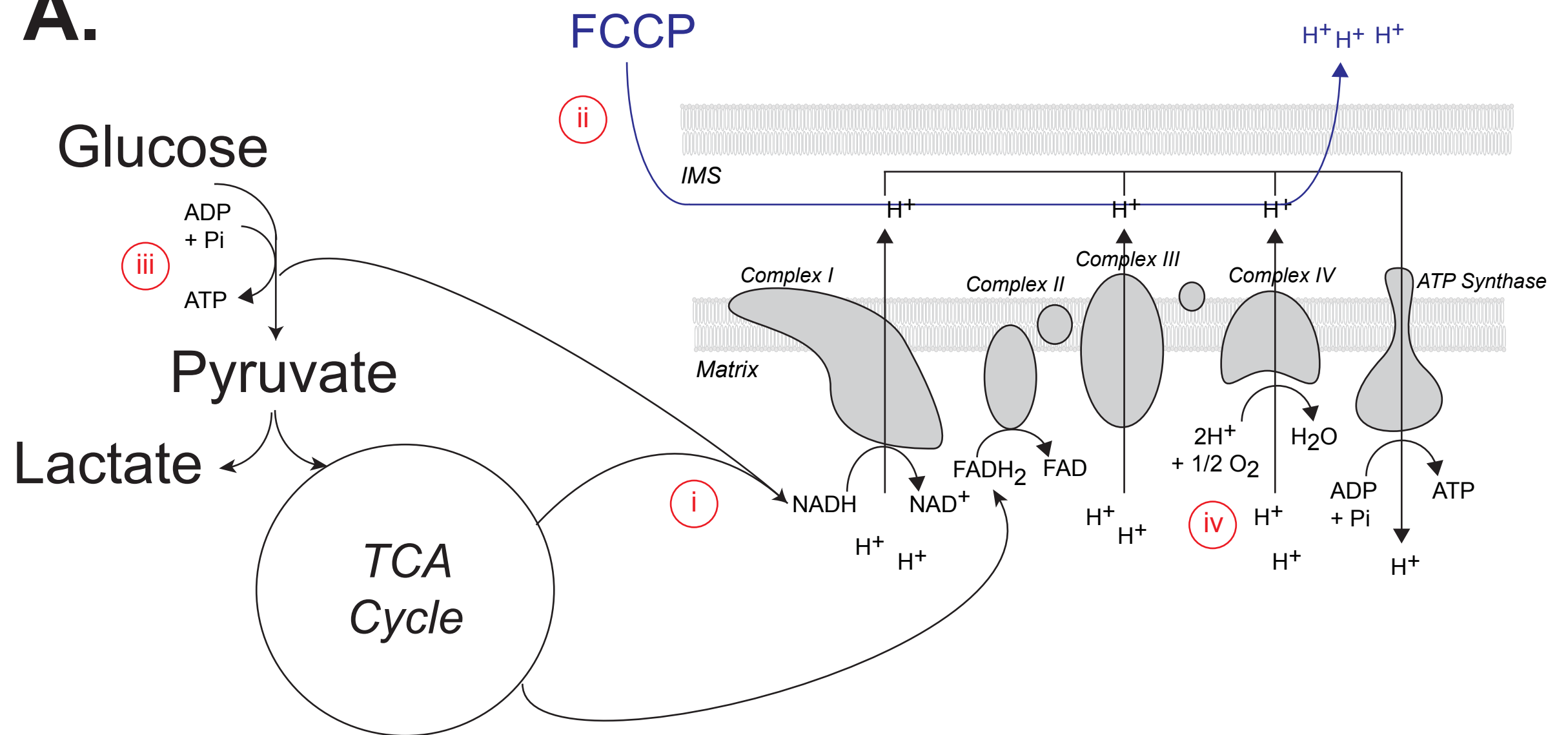

**B.**

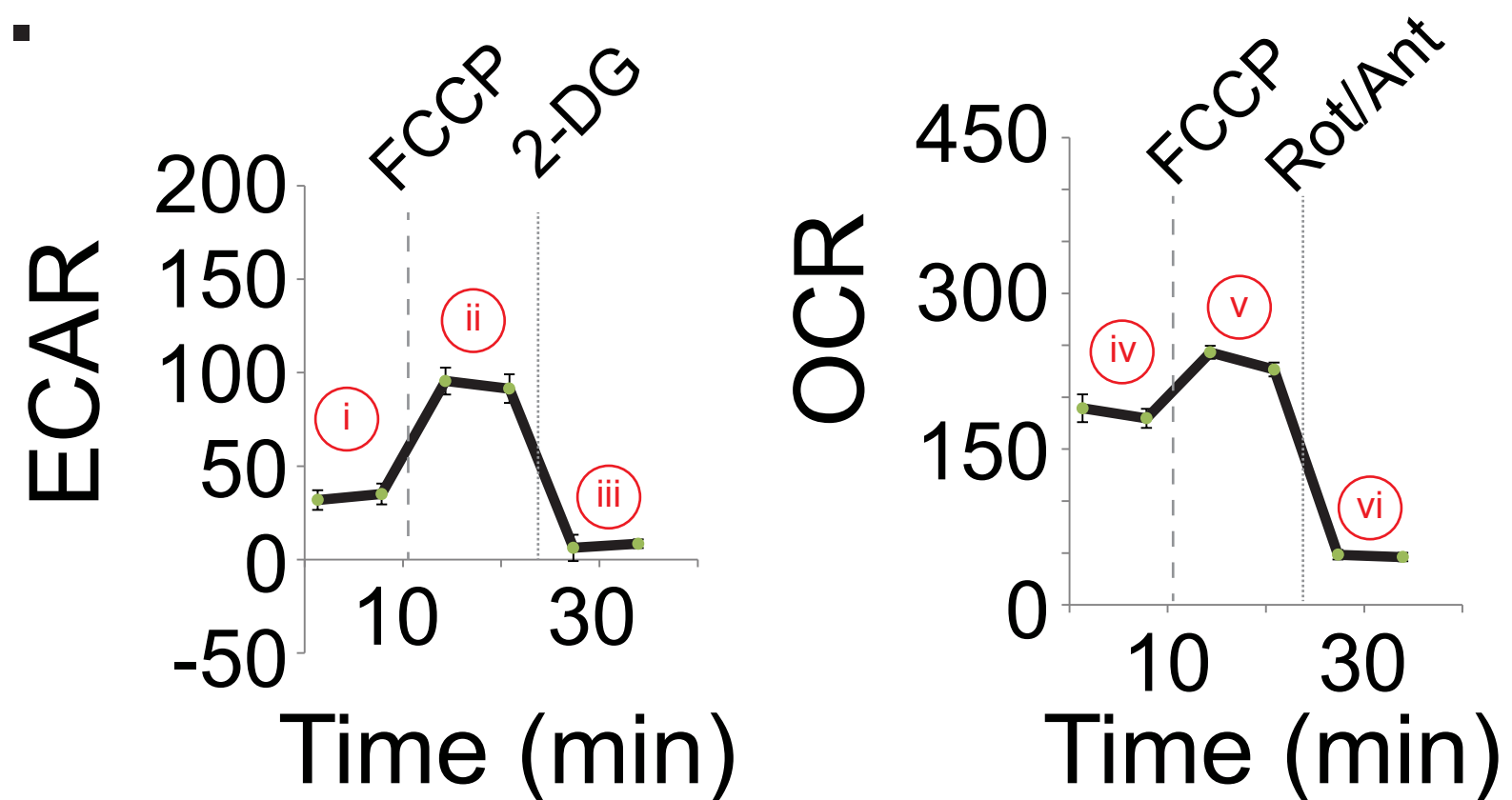

Supplement: Supplementary file 5 — OCR and ECAR measurement explanation. (A) i. Normally functioning cellular respiration utilizes electrons in the form of NADH and FADH2 to pass down the electron transport chain (ETC) gradient. These redox reactions in the ETC pump hydrogen ions from the mitochondrial matrix into the mitochondrial inner membrane space (IMS), providing an electrochemical gradient which in turn powers ATP synthase-dependent ATP production. ii. FCCP is a lipid-soluble ionophore that allows hydrogen ions to escape the IMS, functionally uncoupling the ETC from ATP synthase-mediated ATP production. iii. In order to restore FCCP-mediated depletion of ATP levels, glycolytic flux increases to maximum capacity. iv. In order to maintain a minimal hydrogen ion gradient in the IMS, mitochondrial complex IV activity increases to maximum capacity, thus inducing maximum oxygen consumption. (B) The order of metabolic rate measurements and metabolic toxin treatment for maximal rate measurements. i. Basal glycolytic rate. ii. Respiration arrest-induced maximal glycolytic rate. iii. Glycolytic arrest. iv. Basal OXPHOS rate. v. Depolarization-induced maximal OXPHOS rate. vi. OXPHOS arrest. (PDF 997 kb) [file 40170_2017_168_MOESM5_ESM.pdf]
