# Supplementary material for: Metabolic profiling of triple-negative breast cancer cells reveals metabolic vulnerabilities
Source: Cancer Metab. 2017 Aug 22;5:6. doi: 10.1186/s40170-017-0168-x (PMC5568171; doi:10.1186/s40170-017-0168-x)
Supplement: Supplementary file 7 — Sensitization to metabolic pathway perturbation. (A) Graphical representation of the proportion which each metabolic category (Table S2) was represented in the top 10% sensitivity index scores. (B) The combined top ten pathways as defined by high-scoring sensitivity index genes for each TKI (INC80 or erlotinib). (PDF 801 kb) [file 40170_2017_168_MOESM7_ESM.pdf]

Figure S3

**A**

## Metabolic Category Sensitization

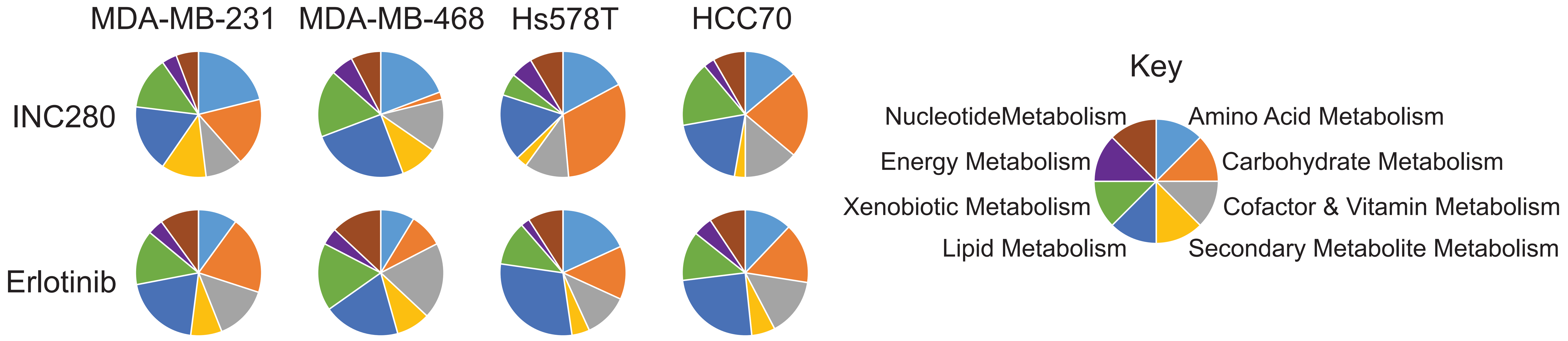


**B**

## Metabolic Pathway Sensitization

| INC280 | |
|---|---|
| **Pathway** | **Combined Hits** |
| Cytochrome P450 Metabolism | 17 |
| Glutathione Metabolism | 17 |
| Tryptophan Metabolism | 16 |
| Drug Metabolism (non-P450) | 13 |
| Fatty Acid Metabolism | 13 |
| Pyrimidine Metabolism | 11 |
| Valine, Leucine, and Isoleucine Degradation | 11 |
| Androgen and Estrogen Metabolism | 9 |
| Arachidonic Acid Metabolism | 9 |
| Purine Metabolism | 9 |

| Erlotinib | |
|---|---|
| **Pathway** | **Combined Hits** |
| Fatty Acid Metabolism | 16 |
| Pyrimidine Metabolism | 16 |
| Cytochrome P450 Metabolism | 15 |
| Glutathione Metabolism | 14 |
| Glycerophospholipid Metabolism | 14 |
| Purine Metabolism | 13 |
| Arachidonic Acid Metabolism | 12 |
| Drug Metabolism (non-P450) | 12 |
| Ether Lipid Metabolism | 11 |
| Nicotinate and Nicotinamide Metabolism | 11 |
